# Zinc bioavailability in rats fed a plant-based diet: a study of fermentation and zinc supplementation

**DOI:** 10.3402/fnr.v59.27796

**Published:** 2015-11-30

**Authors:** Claudia E. Lazarte, Mirian Vargas, Yvonne Granfeldt

**Affiliations:** 1 Department of Food Technology, Engineering and Nutrition, Lund University, Lund, Sweden; 2 Food and Natural Products Center, San Simon University, Cochabamba, Bolivia

**Keywords:** zinc bioavailability, fermentation, phytate, plant-based diets, Wistar rats

## Abstract

**Background:**

Zinc deficiency is a significant problem, in developing countries and in vegetarians, which can be caused by plant-based diets. Thus, dietary strategies, such as fermentation, to improve zinc bioavailability of diets should be investigated.

**Objective:**

To improve zinc bioavailability in a plant-based diet by the inclusion of fermented food.

**Design:**

Cassava tubers were fermented and made to replace the unfermented cassava in a basal plant-based diet, and compared with plant-based diets with and without zinc supplement. The zinc bioavailability of the diets was evaluated in Wistar rats that were fed these diets for 28 days. The evaluation was for zinc apparent absorption (ZnAA), serum zinc levels, and zinc deposits in liver and femur; in addition, the feed efficiency ratio (FER) of the diets and femur weight (FW) of the rats were evaluated.

**Results:**

During the cassava fermentation, lactic acid increased and pH decreased (from 6.8 to 3.9), which is favorable for native phytase activity, resulting in a 90.2% reduction of phytate content in cassava. The diet containing fermented cassava showed significantly higher levels of ZnAA, FER, and FW (*p<*0.001). Moreover, the zinc levels in serum and femur were significantly higher (*p<*0.001) compared with the results of the diet with unfermented cassava. The results clearly show a higher zinc bioavailability in the diet containing fermented cassava and are comparable with the results obtained with the plant-based diet with zinc supplement.

**Conclusions:**

In conclusion, the fermentation of cassava reduces the phytate content. The diet containing the fermented cassava represents a better nutritional alternative than the diet with unfermented cassava and is comparable with the zinc-supplemented diets.

Zinc is an essential micronutrient for human growth, for cognitive development, and for maintenance of the immune system. Zinc deficiency is a serious global problem, particularly in developing countries; vegetarians are also affected. Deficiencies arise from an inadequate intake or reduced absorption of zinc from the diet. This impairs the immune response, which may lead to an increased susceptibility to infections and diseases (1, 2). Nowadays, a variety of nutritional strategies are applied to reduce the occurrence of zinc deficiency; among these are supplementation, fortification, and dietary modification for enhancing the bioavailability of zinc (3, 4).

Studies involving micronutrient supplements or fortification have been carried out in developing countries. However, in many cases, the results were disappointing, as no effect of zinc supplementation was observed on growth. One of the given reasons for the lack of success was the plant-based, high-phytate diets, which may produce perturbations on the zinc homeostasis because of the limited zinc bioavailability. Perturbations such as reduced endogenous secretion of zinc, low absorption of zinc, and excessive endogenous fecal zinc were reported (5–7). Moreover, supplementation with micronutrients is not a long-term solution and its sustainability will always rely on stable financial and technical support, which is difficult to maintain in developing countries. Thus, a more convenient approach for rural areas, where the diets are plant-based, is to use dietary strategies to enhance the bioavailability of multiple micronutrients. In previous studies in a tropical rural area in Bolivia, we reported the main components of the diet; tubers and cereals with only a small contribution of animal source food, constituting a plant-based diet, likely high in phytates and low in zinc absorption (8, 9).

Phytate (phytic acid or myo-inositol-6-phosphate IP6) can bind divalent minerals, preventing their absorption and utilization by the body. The inhibitory effect of phytate appears to follow a dose-dependent response, the molar ratios phytate:mineral are used as an indication of the absorbable mineral. Molar ratios phytate:zinc (Phy:Zn) between 5 and 15 may have a certain negative effect on the absorption of zinc, and molar ratios higher than 15 clearly inhibit zinc absorption (2). The desirable ratios for phytate:iron (Phy:Fe) are <1 (10), and for phytate:calcium (Phy:Ca) <0.17 (11). Fermentation is an advised process for reducing the level of phytate in food, by the activation of the endogenous native phytases and production of microbial phytase, which degrades phytates by the successive removal of the phosphate groups, resulting in an increased mineral bioavailability (3, 12, 13). There are studies showing the increased mineral bioavailability in fermented food *in vitro* (14, 15) and a few *in vivo* studies in rats fed fermented food (16). However, to our knowledge, *in vivo* studies of mineral bioavailability in rats fed plant-based diets containing fermented food are still limited.

The objective of the present paper was to improve zinc bioavailability in a plant-based diet (consisting of cassava, rice, plantain, and egg) commonly consumed in the rural tropical area Chapare, Bolivia. Thus, cassava was fermented and made to replace the unfermented cassava in the basal plant-based diet (BPBD), constituting a modified plant-based diet (MPBD). *In vivo* biological evaluation of the diets was conducted in Wistar rats, and the results were compared with zinc-supplemented plant-based diets (BPBD+15 and BPBD+30).

## Materials and methods

### Processing of cassava tubers

Cassava (*Manihot esculenta*) is a tuber widely consumed in tropical areas around the world, and it was selected for fermentation. The fermentation procedure was performed in duplicate under laboratory conditions, following the usual fermentation process carried out in some tropical areas in Bolivia. The cassava tubers from the local market at Chapare, Bolivia, were processed to obtain two products: boiled cassava flour as an ingredient for the BPBD and toasted fermented cassava flour for the MPBD. Raw cassava tubers were peeled, washed, and divided into two parts; one part was chopped into pieces (approx. 10×5×3 cm) and boiled (15 min at 93°C). Thereafter, the boiled cassava was dried for 12 h at 60°C (heating oven; model ED23, Binder, Germany) and ground in a mill (Grinder Moulinette, Moulinex, Brazil) into cassava flour for preparation of the BPBD. The other part was used for fermentation; cassava was grated (approx. 50×5×3 mm) and placed into an airtight plastic container with sufficient distilled water to cover the grated cassava.

The fermentation proceeded spontaneously for a period of 14 days at room temperature (20–25°C). Each day during the process, a sample was extracted to evaluate changes in pH, acidity as lactic acid content (mg lactic acid/100 g sample), and phytate content. After 14 days, the bulk of fermented cassava was transferred into cloth bags and pressed manually to remove most of the water, dried for 12 h at 60°C, ground, toasted for 15 min in a stainless steel pan, and stored for the preparation of the MPBD.

### Diets

The food items that are most commonly consumed and produced in Chapare were previously identified by a food frequency questionnaire (17). It was in accordance with these results that the food components of the BPBD were selected (i.e. cassava, rice, plantain, and eggs) and purchased in local markets in Chapare.

The plantains, rice, and eggs were boiled individually for approximately 20 min at 93°C until the tissue was soft, dried for 12 h at 60°C, ground, and finally mixed with the boiled cassava flour. The BPBD was prepared by mixing cassava flour (42%), rice flour (40%), plantain (5%), and egg powder (13%). The MPBD was prepared by mixing the same percentages of the ingredients and replacing boiled cassava flour by fermented cassava flour. In addition, in order to elucidate how the matrix of the diets affect zinc absorption when zinc is supplemented, the BPBD was supplemented with zinc; 15 and 30 µgZn/g diet were added to the BPBD (BPBD+15 and BPBD+30). All of the diets were formulated to meet the growth requirements of the rats for protein (10–12 g protein/100 g diet), energy (1,600–2,000 kJ/100 g diet), and zinc (1.2–2.5 mg/100 g diet) (18).

### Animals and biological assay

The biological assay was based on the methodology to evaluate zinc bioavailability in rats (16, 19, 20). A total of 36 six-week-old Wistar rats (six rats *per* diet), weighing 100±5 g, were selected and housed individually in metabolic cages at 21±2°C, with alternating 12-h light–dark cycles. The diets were fed *ad libitum*; each day the amount consumed was weighed and recorded as well as the weight of the animal. The feeding test proceeded for 28 days, with free access to water. During the last 7 days, the feces were collected, weighed, and recorded individually for further zinc analysis. Urinary excretion, which is another way by which zinc is lost from the body, was not determined in the present research because previous studies indicated that urinary excretion does not significantly vary in response to dietary zinc intake, and it accounts for only 1–3% of the amount of zinc excreted in feces (21, 22). At the end of the experiment, the rats underwent cardiac puncture under anesthesia. The thorax was opened, blood was drawn into heparinized tubes and separated into serum, for zinc analysis. The livers and right femurs were removed, cleaned of adhering tissue, weighed, dried, and stored for further zinc analysis. The feed efficiency ratio (FER) of the diets was calculated by dividing body weight gain (BWG) by food intake. Femur weight (FW) was recorded as growth parameter. Zinc apparent absorption (ZnAA) was evaluated with the zinc intake and excretion. Serum zinc levels and the zinc deposits in the liver and femur were used as markers of zinc bioavailability (16, 19, 20).

### Chemical analyses

Protein, fat, and fiber were determined by official methods of analysis (AOAC 14.026, AOAC 14.019, and AOAC 14.020, respectively) (23). Starch was determined by the enzymatic method (24) and carbohydrates and energy were calculated.

Zinc, iron, and calcium content in dry individual food items, diets, feces, liver, femur, and serum of the rats were quantified by flame atomic absorption spectrometry (Model AAnalyst 200, Perkin Elmer Corporation, Norwalk, CT) (25). Before analysis, the solid samples were acid digested in a microwave reaction system (Model Multiwave PRO, Anton Paar Co., Ashland, USA). Certified reference materials for trace elements BCR^®^ (rice flour IRMM 804 FLUKA and bovine liver BCR185R FLUKA Sigma-Aldrich Co., St. Louis, USA) were used to validate the mineral analysis in solid samples. The reference material Seronorm™ (serum L-1-2. SERO AS, Norway) was used to validate the zinc analysis in serum samples.

Phytate was quantified as inositol phosphate IP-6 in cassava, in fermented cassava, and in diets by high-performance ion chromatography (HPIC) (Guard Column Dionex corp., Sunnyvale, CA. HIPC CarboPac PA-100 250×4 mm id, Waters 486, tunable absorbance detector), according to the method described by Carlsson et al. (26). Briefly, dry samples were acid extracted under magnetic stirring, frozen overnight, thawed, and centrifuged at 12,000 g for 10 min; the supernatants were decanted and injected to HPIC system with post-column reactor (Fe(NO_3_)_3_·9H_2_O); and absorbance was monitored at 290 nm using UV detection (Waters 486, tunable absorbance detector).

### Statistical analysis

The normality of the data was evaluated by Shapiro-Wilk test; the data followed a normal distribution. Statistical analysis for the nutritional changes in cassava through fermentation was performed by *t*-paired test. The nutritional differences between the four diets and differences between the results of the biological assay were evaluated by one-way ANOVA. Where significant effects were found, *post-hoc* analysis was computed and differences between means were assessed by Tukey test. Correlations and linear regression analysis were performed to evaluate associations during fermentation and changes in femur and serum zinc because of the intake of zinc and Phy:Zn. Statistical package for Social Sciences v.18.0 (SPSS Inc., IBM Corporation 2010, www.spss.com) was used, the significance level was set up at *p*<0.05.

## Results

### Processing of cassava tubers fermentation

Fiber, fat, starch, and calcium showed small differences between fermented and raw cassava. Differences in protein, zinc, and iron content were significant at level *p*<0.05; the values were somewhat lower in the fermented cassava, probably because of these components being leaked into the water during fermentation (Table 1).

**Table 1:** Effect of cassava fermentation on nutrient content, in dry weight (*n=*4)

	Raw	Fermented		
				
Nutrient	Mean±SEM	Mean±SEM	% Difference^a^	*p*
Protein (g/100 g)	1.62±0.015	1.42±0.010	−12.1	0.016*
Fat (g/100 g)	0.44±0.020	0.57±0.015	28.4	0.174
Fiber (g/100 g)	2.45±0.015	2.36±0.030	−3.48	0.310
Starch (g/100 g)	78.9±1.02	67.7±0.75	−14.1	0.100
Zinc (mg/100 g)	1.66±0.005	1.60±0.004	−3.83	0.015*
Iron (mg/100 g)	0.56±0.012	0.54±0.011	−3.39	0.017*
Calcium (mg/100 g)	53.4±0.84	52.1±0.67	−2.35	0.558
Phytate (mg/100 g)	273.1±2.65	26.9±0.65	−90.2	0.005**
Molar ratios				
Phy:Zn	16.31±0.204	1.71±0.004	−89.5	0.009**
Phy:Fe	41.30±0.501	4.31±0.090	−89.6	0.007**
Phy:Ca	0.310±0.002	0.032±0.000	−89.7	0.005**

* Significant difference at level *p<*0.05 (paired *t*-test).

** Significant difference at level *p<*0.01.

a % Difference between fermented cassava (FCa) and raw cassava (RCa), calculated as % Difference=(FCa−RCa)*100/RCa.

**Fig. 1:**
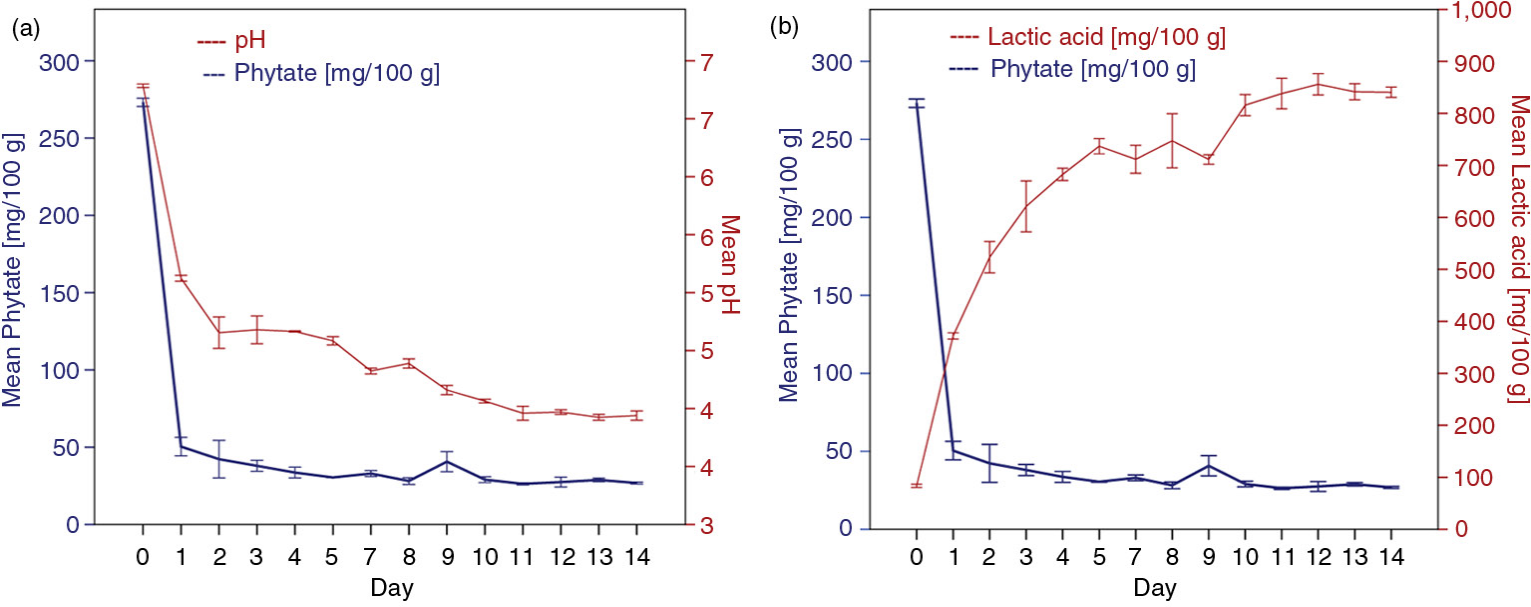
Changes of phytate with (a) pH and (b) lactic acid content through fermentation of cassava.

Initial and final values of pH, lactic acid, and phytate were significantly different (*p*<0.001). The pH decreased from 6.80 to 3.95, as a consequence of lactic acid production which increased from 84 to 841 mg lactic acid/100 g (Fig. 1). Phytate was reduced by 90.2%, which significantly decreased the ratios of Phy:Zn, Phy:Fe, and Phy:Ca; all of which were reduced by about 89% (Table 1).

Phytate was positively correlated with pH (*r=*0.917, *p*<0.001); the linear regression coefficient (*β*=80.61, *p*<0.001) indicated that phytate decreased by 80.61 mg/100 g for every unit reduced in pH, until they reached constant values. A negative correlation of phytate with lactic acid concentration was found (*r=*−0.958, *p*<0.001). The regression coefficient (*β*=−0.256, *p*<0.001) indicated that phytate decreased by 0.256 mg/100 g for every additional mg/100 g of lactic acid.

### Composition of the diets

Phytate content in the MPBD was significantly lower than in BPBD, BPBD+15, and BPBD+30 (Table 2). The Phy:Zn was significantly different between the diets, with the following order BPBD (7.79)>BPBD+15 (4.43)>BPBD+30 (3.20)≈MPBD (2.76), indicating that zinc relative bioavailability was the highest in MPBD which was comparable to BPBD+30, and the lowest was in BPBD. The Phy:Fe and Phy:Ca indicate that the relative bioavailability of iron and calcium was the highest in MPBD and the lowest in BPBD (Table 2).

**Table 2:** Composition of the diets in dry weight

	BPBD	BPBD+15	BPBD+30	MPBD
Energy (kJ/100 g)	1,762±7.7^a^	1,762±7.7^a^	1,762±7.7^a^	1,774±1.1^a^
Protein (g/100 g)	9.75±0.055^a^	9.75±0.055^a^	9.75±0.055^a^	10.02±0.085^a^
Fat (g/100 g)	5.5±0.40^a^	5.5±0.40^a^	5.5±0.40^a^	5.9±0.08^a^
Fiber (g/100 g)	2.16±0.040^b^	2.16±0.040^b^	2.16±0.040^b^	1.56±0.030^b^
Carbohydrates (g/100 g)	83.1±0.39^b^	83.1±0.39^b^	83.1±0.39^b^	82.5±0.19^b^
Zinc (mg/100 g)	1.99±0.040^a^	3.49±0.038^b^	4.84±0.185^c^	1.94±0.057^a^
Iron (mg/100 g)	1.14±0.043^b^	1.14±0.043^b^	1.14±0.043^b^	1.33±0.062^b^
Calcium (mg/100 g)	50.7±1.30^a^	50.7±1.30^a^	50.7±1.30^a^	57.5±8.97^a^
Phytic acid (mg/100 g)	156.2±0.86^c^	156.2±0.86^c^	156.2±0.86^c^	54.0±0.00^b^
Molar ratios				
Phy:Zn	7.79±0.199^d^	4.43±0.024^c^	3.20±0.105^b^	2.76±0.029^b^
Phy:Fe	11.58±0.373^c^	11.58±0.373^c^	11.58±0.373^c^	3.44±0.096^b^
Phy:Ca	0.187±0.004^c^	0.187±0.004^c^	0.187±0.004^c^	0.058±0.008^b^

BPBD, basal plant-based diet; BPBD+15, basal plant-based diet with 15 µg/g zinc supplement; BPBD+30, basal plant-based diet with 30 µg/g zinc supplement; MPBD, modified plant-based diet (containing fermented cassava). Results presented as mean±SEM (*n*=2);

a–d Indicate that the values within a row, for each variable, that are not sharing the same superscript letter were significantly different (*p*< 0.05) (ANOVA analysis).

### Biological assay

Results from the biological assay show that replacement of cassava with fermented cassava in the BPBD as well as the addition of zinc supplement had a positive effect on FER, FW, and ZnAA. These parameters were calculated for the four diets (Table 3); the BPBD resulted in a significantly lower FER and FW in the rats compared with rats fed the other diets. However, when fermented cassava flour replaced the plain cassava flour in the MPBD, the differences of BWG and FW were no longer significant compared with zinc-supplemented diets (BPBD+15 and BPBD+30). Thus, the addition of fermented cassava on the BPBD or the addition of zinc supplement on the BPBD significantly increased the growth parameters FER and FW.

**Table 3:** Effect of different diets on food efficiency ratio, femur weight, apparent zinc absorption, zinc in serum, and zinc retention in liver and femur of rats (*n=*6) (DW)

Group	FER	FW (g)	ZnAA%	Zn in liver (µg/g)	Zn in femur (µg/g)	Zn in serum (µg/dl)
BPBD	0.16±0.008^a^	0.55±0.009^a^	16.5±1.43^a^	88±2.1^a^	143.8±5.4^a^	107±4.8^a^
BPBD+15	0.24±0.025^b^	0.68±0.020^bc^	45.5±1.88^b^	95±3.9^a^	207.9±15.0^bc^	190±6.4^bc^
BPBD+30	0.31±0.019^bc^	0.78±0.027^cd^	57.3±1.63^c^	102±6.3^a^	267.3±19.5^cd^	211±7.5^c^
MPBD	0.27±0.010^b^	0.77±0.031bcd	40.2±0.79^b^	100±3.8^a^	186.7±6.6^ab^	161±5.3^b^
P*	<0.001	<0.001	<0.001	<0.001	<0.001	<0.001

FER, feed efficiency ratio, calculated as BWG/food intake; FW, relative femur weight; ^3^ZnAA, Zinc apparent absorption.

* ANOVA analysis, significant at level *p<*0.001.

a–d indicate that the values within a column for each variable that are not sharing the same superscript letter were significantly different (*p*< 0.05). DW, dry weight; BPBD, basal plant-based diet; BPBD+15, basal plant-based diet with 15 µg/g zinc supplement; BPBD+30, basal plant-based diet with 30 µg/g zinc supplement; MPBD, modified plant-based diet (containing fermented cassava).

**Fig. 2:**
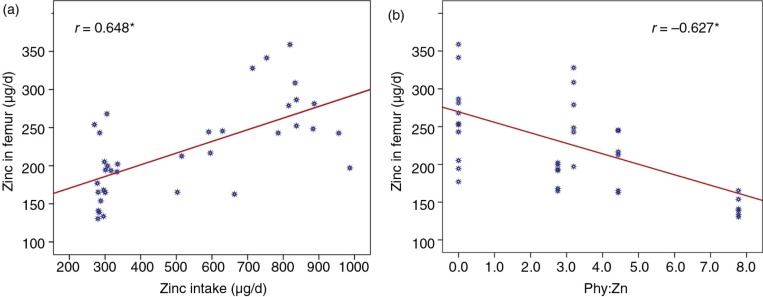
Zinc in femur as indicator of zinc bioavailability: (a) correlation with zinc intake and (b) correlation with Phy:Zn intake. *Significant correlations, *p*=0.01.

Results of ZnAA, zinc in serum, and zinc retention in liver and femur of the rats (Table 3) indicate a very low zinc absorption of 16.5% for BPBD, and the lowest values of zinc in serum, femur, and liver. ZnAA was significantly lower in BPBD than after all the other diets, as well as serum zinc and zinc in femur, except for zinc in femur after the MPBD. Results of the diet with fermented cassava (Table 3) show comparable values of all the parameters to those obtained with the zinc-supplemented diet BPBD+15, except for a lower value of zinc in serum. The zinc intake was positively correlated with the retention of zinc in femur (*r=*0.648, *p*=0.01) (Fig. 2a) and with the zinc in serum (*r=*0.697, *p*=0.01). A linear regression analysis showed a direct association between the zinc in femur and the zinc intake (*β*=0.153, *p*<0.001), zinc in femur increased by 0.153 µg/d for every additional µg/d of zinc intake. Zinc in femur was correlated with the ZnAA (*r=*0.800, *p*<0.01). Furthermore, zinc in femur was negatively correlated with the molar ratio of Phy:Zn (*r=*−0.627, *p*< 0.01) (Fig. 2b), with an inverse association (*β*=−13.891, *p*<0.001), indicating that zinc in femur decreased by 13.891 µg/d for every additional unit of Phy:Zn. A similar trend was seen in serum zinc (*r=*−0.757, *p*<0.01).

## Discussion

The most important finding in the present study was that the very low absorption of zinc (16.5%) in a BPBD increased over 240% when cassava was fermented before preparation of the diet. After the diet containing fermented cassava, the absorption level of zinc (40.2%) was statistically comparable with the level of the basal diet with zinc supplement BPBD+15 (45.5%). The levels of zinc in serum, zinc in femur, FER, and FW were also higher after the BPBD. During cassava fermentation, 90.2% of phytate level was decreased. Consequently, the phytate:mineral ratios which before fermentation were at levels likely to inhibit the absorption of Zn, Fe, and Ca were significantly decreased. The results indicate that cassava fermentation increases the theoretical bioavailability of Zn, Fe, and Ca in cassava, and the experimental zinc bioavailability evaluated in rats fed the diet containing the fermented cassava.

This improvement on mineral bioavailability in the diet containing fermented cassava was mainly due to the reduction of phytate during the fermentation, which was attributed to the lactic acid production and pH reduction. After 24 h of fermentation, the lactic acid content increased four times and pH decreased from 6.8 to 5.1, indicating a positive correlation with the phytates reduction, in agreement with previous studies of cassava fermentation (27–29). The reduction of pH is shown to be a favorable condition for the native phytase activity, plant phytases have optimum pH at 4.8–5.6 and their activities varied markedly according to the pH. Previous studies have shown an optimum pH to activate the endogenous phytase in cereals, plant grains, and seeds to be in the interval from 4.5 to 5.5 (12, 30). Türk and Sandberg (31) reported a reduction of 64% of phytate at pH 5.7 after 150 min of spontaneous wheat fermentation. In the present study, after 24 h of spontaneous cassava fermentation, phytate was reduced in 81.5%, and by the end of the fermentation (14 days), the reduction reached 90.2% and the pH decreased to 3.9.

In addition to the optimum pH conditions for the native phytase activity, it is also probable that the reduction of phytates was because of the phytase elaborated with *Lactobacillus* bacteria through the lactic fermentation. Negative correlation of phytate with lactic acid level was found (*r=*−0.958, *p*<0.001). Previous studies have shown that through cassava fermentation, there is an increasing production of organic acids – mainly lactic acid and bacteria primarily of the strain *Lactobacillus* are also produced (29, 32). Previous studies reported that bacteria *Lactobacillus* isolated during spontaneous fermentation of maize, soybean, and other cereals was able to break down phytates. Thus, the capability of lactic acid bacteria to hydrolyze phytates by the production of the phytase was established (27, 33). The optimal pH for the production of phytase was found to be between 4.5 and 6 (30, 33, 34). In that pH range, *Lactobacillus* bacteria were able to degrade 30% of phytates in 2 h during wheat flour fermentation (35).

The Phy:Fe and Phy:Ca indicated that the relative bioavailability of iron and calcium was the highest in MPBD and the lowest in BPBD. Regarding Phy:Zn ratio, it was significantly different between the diets. Thus, the main difference between the diets BPBD and MPBD was the phytate content; the BPBD had a Phy:Zn 2.8 times higher than the MPBD. Previous studies have reported that in phytate-rich diets, the bioavailability of trace elements, such as zinc, calcium and iron, is highly depressed in humans and animals (2, 6). The BPBD was composed of the main food consumed in the population of the tropical area in Bolivia, a diet with low zinc absorption (8); hence, dephytinization strategies such as fermentation are highly recommended in this area.

Results from the biological assay showed that replacement of cassava with fermented cassava in the BPBD, and the addition of zinc supplement, had a positive effect on FER, FW, and ZnAA (Table 2); rats fed BPBD showed lower FER and FW compared with rats fed the other diets with lower levels of phytate. However, when fermented cassava flour replaced the plain cassava flour in the MPBD, the differences of FER and FW were no longer significant compared with BPBD+15 and BPBD+30. FW was lower in the group after BPBD (3.50±0.12 g/kgBW) than after MPBD (3.56±0.11 g/kgBW) and not different to after zinc-supplemented diets. Therefore, the results indicated that MPBD and zinc-supplemented diets not only increased bone weight but also fat and muscle tissue. This is in agreement with previous researchers, who reported that diets with high Phy:Zn showed a significant depression of growth in rats and can lead to typical symptoms of zinc deficiency (36, 37). In our study, besides the lower FER and FW, no symptoms of zinc deficiency were recorded in the rats fed BPBD indicating that the phytate content in this diet may decrease the growth rate, but it was not at levels to induce zinc deficiency during the 28 days of the assay.

Furthermore, the levels of ZnAA were positively correlated to zinc in serum (*r=*0.903, *p*=0.01) and zinc retention in femur (*r=*0.800, *p*=0.01). ZnAA levels were significantly lower in rats fed BPBD compared with those obtained in rats fed MPBD or zinc-supplemented diets (BPBD+15 and BPBD+30) (Table 3). With regard to zinc in femur, it was observed that the MPBD had a positive effect, which was consistent with the higher absorption of zinc, and was equivalent to the effect of BPBD with either level of zinc supplement (15 or 30 µg/g). The levels of zinc in liver after the different diets were not significantly different; similarly, no differences were found in previous studies of improvement of zinc bioavailability (38).

The zinc intake was positively correlated with the retention of zinc in femur (*r=*0.648, *p*=0.01) (Fig. 2a) and with the zinc in serum (*r=*0.697, *p*=0.01). Zinc in femur was also correlated with the ZnAA (*r=*0.800, *p*<0.01). Hence, the level of zinc in femur is a good indicator of zinc bioavailability in rats, as shown by other authors (16, 19, 39). The amount of phytate in the diets has negatively affected the ZnAA, the level of zinc in femur (Fig. 2b), and serum in rats fed BPBD. This might be explained by previous findings (19, 37), which showed that phytate acts as a binding agent between zinc and other minerals, reducing their solubility and bioavailability. Moreover, it was shown that phytate not only reduces the dietary zinc absorption but it also reduces the reabsorption of endogenous intestinal zinc (19, 37). In studies with rats fed diets supplemented with phytate (0.5–1.0%), a reduction in the ZnAA from 52 to about 25% (37) has been shown. In agreement with previous studies, in the present research, we found inverse correlation between the phytate content and ZnAA (*r=*−0.948, *p*=0.001) with the non-supplemented diets. ZnAA ranged from 44.5 down to 16.5% when the rats were fed diets with phytate concentration from 54 mg/100 g (0.05%) to 156.2 mg/100 g (0.15%).

## Conclusions

The present paper evidences that fermentation increases the theoretical bioavailability of Zn, Fe, and Ca in cassava, besides increasing the experimental zinc bioavailability evaluated in rats fed a diet containing fermented cassava. Thus, fermentation can be an efficient dephytinization strategy. The inclusion of fermented cassava in a plant-based diet represented a better nutritional alternative than the diet with unfermented cassava, with comparable results from the plant-based diet supplemented with zinc. Besides, cassava fermentation may be a more economical alternative than the use of supplements and can therefore be advantageous both in practical and economical terms. This is conducive to the idea of sustainability, not only for this specific population but also for many others in developing countries, where animal sources of food are limited.

## References

[1] Prasad AS (2000). Effects of zinc deficiency on immune functions. J Trace Elem Exp Med.

[2] Hotz C, Brown KH (2004). International Zinc Nutrition Consultative Group (IZiNCG) technical document #1. Assessment of the risk of zinc deficiency in populations and options for its control. Food Nutr Bull.

[3] Gibson RS, Hotz C (2001). Dietary diversification/modification strategies to enhance micronutrient content and bioavailability of diets in developing countries. Br J Nutr.

[4] Grewal HK, Hira CK (2003). Effect of processing and cooking on zinc availability from wheat (Triticum aestivum). Plant Foods Hum Nutr.

[5] Manary MJ, Hotz C, Krebs NF, Gibson RS, Westcott JE, Broadhead RL (2002). Zinc homeostasis in Malawian children consuming a high-phytate, maize-based diet. Am J Clin Nutr.

[6] Lopez HW, Leenhardt F, Coudray C, Remesy C (2002). Minerals and phytic acid interactions: is it a real problem for human nutrition?. Int J Food Sci Tech.

[7] Mazariegos M, Hambidge KM, Westcott JE, Solomons NW, Raboy V, Das A (2010). Neither a zinc supplement nor phytate-reduced maize nor their combination enhance growth of 6- to 12-month-old Guatemalan infants. J Nutr.

[8] Lazarte C, Encinas ME, Alegre C, Granfeldt Y (2012). Validation of digital photographs, as a tool in 24-h recall, for the improvement of dietary assessment among rural populations in developing countries. Nutr J.

[9] Lazarte C, Alegre C, Rojas E, Granfeldt Y (2013). Nutritional status of patients with cutaneous leishmaniasis from a tropical area from Bolivia, and implications for zinc bioavailability. Food Nutr Sci.

[10] Hurrell RF (2004). Phytic acid degradation as a means of improving iron absorption. Int J Vitam Nutr Res.

[11] Umeta M, West CE, Fufa H (2005). Content of zinc, iron, calcium and their absorption inhibitors in foods commonly consumed in Ethiopia. J Food Compos Anal.

[12] Reale A, Konietzny U, Coppola R, Sorrentino E, Greiner R (2007). The importance of lactic acid bacteria for phytate degradation during cereal dough fermentation. J Agric Food Chem.

[13] Poutanen K, Flander L, Katina K (2009). Sourdough and cereal fermentation in a nutritional perspective. Food Microbiol.

[14] Hemalatha S, Platel K, Srinivasan K (2006). Influence of germination and fermentation on bioaccessibility of zinc and iron from food grains. Eur J Clin Nutr.

[15] Bergqvist SW, Andlid T, Sandberg A-S (2006). Lactic acid fermentation stimulated iron absorption by Caco-2 cells is associated with increased soluble iron content in carrot juice. Br J Nutr.

[16] Tesan FC, Collia N, Arnoldi S, Fuda J, Torti H, Weill R (2009). Relative bioavailability of zinc in yogurt using body weight gain, femur weight and bone zinc content in rats as markers. Open Nutraceuticals J.

[17] Lazarte CE, Carlsson N-G, Almgren A, Sandberg A-S, Granfeldt Y (2015). Phytate, zinc, iron and calcium content of common Bolivian food, and implications for mineral bioavailability. J Food Compos Anal.

[18] NAP (1995). Nutrient requirements of laboratory animals.

[19] McClung JP, Stahl CH, Marchitelli LJ, Morales-Martinez N, Mackin KM, Young AJ (2006). Effects of dietary phytase on body weight gain, body composition and bone strength in growing rats fed a low-zinc diet. J Nutr Biochem.

[20] Ahmed A, Anjum F, Randhawa M, Farooq U, Akhtar S, Sultan M (2012). Effect of multiple fortification on the bioavailability of minerals in wheat meal bread. J Food Sci Technol.

[21] Kirchgessner M (1980). Total true efficiency of zinc utilization: determination and homeostatic dependence upon zinc supply status in young rats. J Nutr.

[22] Johnson PE, Hunt JR, Ralston NV (1988). The effect of past and current dietary Zn intake on Zn absorption and endogenous excretion in the rat. J Nutr.

[23] AOAC (1984). Official methods of analysis of the association of official analytical chemists.

[24] Holm J, Björck I, Drews A, Asp NG (1986). A rapid method for the analysis of starch. Starch – Stärke.

[25] Taylor A (1997). Measurement of zinc in clinical samples. Ann Clin Biochem.

[26] Carlsson N-G, Bergman E-L, Skoglund E, Hasselblad K, Sandberg A-S (2001). Rapid analysis of inositol phosphates. J Agric Food Chem.

[27] Freire AL, Ramos CL, de Almeida EG, Duarte WF, Schwan RF (2014). Study of the physicochemical parameters and spontaneous fermentation during the traditional production of yakupa, an indigenous beverage produced by Brazilian Amerindians. World J Microbiol Biotechnol.

[28] Aro SO (2008). Improvement in the nutritive quality of cassava and its by-products through microbial fermentation. Afr J Biotechnol.

[29] Afolabi OR, Popoola TOS (2004). Phytase activity of *Lactobacillus plantarum* strains from fufu-fermented cassava. Trop Sci.

[30] Greiner R, Konietzny U (2009). Phytate-degrading enzymes for food application. New Biotechnol.

[31] Türk M, Sandberg AS (1992). Phytate degradation during breadmaking: effect of phytase addition. J Cereal Sci.

[32] Adewusi SRA, Ojumu TV, Falade OS (1999). The effect of processing on total organic acids content and mineral availability of simulated cassava-vegetable diets. Plant Foods Hum Nutr.

[33] Coban H, Demirci A (2014). Screening of phytase producers and optimization of culture conditions for submerged fermentation. Bioprocess Biosyst Eng.

[34] Oboh G, Akindahunsi AA, Oshodi AA (2003). Dynamics of phytate-zinc balance of fungi fermented cassava products (gari and flour). Plant Foods Hum Nutr.

[35] Lopez HW, Ouvry A, Bervas E, Guy C, Messager A, Demigne C (2000). Strains of lactic acid bacteria isolated from sour doughs degrade phytic acid and improve calcium and magnesium solubility from whole wheat flour. J Agric Food Chem.

[36] Fredlund K, Isaksson M, Rossander-Hulthén L, Almgren A, Sandberg A-S (2006). Absorption of zinc and retention of calcium: dose-dependent inhibition by phytate. J Trace Elem Med Bio.

[37] Rimbach G, Pallauf J, Moehring J, Kraemer K, Minihane AM (2008). Effect of dietary phytate and microbial phytase on mineral and trace element bioavailability – a literature review. Curr Top Nutraceut R.

[38] Shockravi S, Mohammad-Shirazi M, Abadi A, Seyedain M, Kimiagar M (2012). Phytase supplementation improves blood zinc in rats fed with high phytate Iranian bread. J Res Med Sci.

[39] Yonekura L, Suzuki H (2005). Effects of dietary zinc levels, phytic acid and resistant starch on zinc bioavailability in rats. Eur J Nutr.

